# The prevalence and burden of avoidant/restrictive food intake disorder (ARFID) in a general adolescent population

**DOI:** 10.1186/s40337-023-00831-x

**Published:** 2023-06-29

**Authors:** Lara Van Buuren, Catharine Anne Kerle Fleming, Phillipa Hay, Kay Bussey, Nora Trompeter, Alexandra Lonergan, Deborah Mitchison

**Affiliations:** 1 School of Psychology, Western Sydney University, Sydney, Australia; 2 School of Health Sciences, Western Sydney University, Sydney, Australia; 3 Translational Health Research Institute, School of Medicine, Western Sydney University, Sydney, Australia; 4 Centre for Emotional Health, School of Psychological Sciences, Macquarie University, Sydney, Australia

**Keywords:** Avoidant/restrictive food intake disorder, Eating disorder, Prevalence, Burden, Adolescence

## Abstract

**Background:**

Little is known about the prevalence and impairment associated with possible Avoidant/restrictive food intake disorder (ARFID) in community adolescent populations. We aimed to investigate the prevalence, health-related quality of life (HRQoL), and psychological distress associated with possible ARFID in a sample of adolescents from the general population in New South Wales, Australia.

**Methods:**

A representative sample of 5072 secondary school students aged between 11 and 19 years completed the online EveryBODY survey in 2017. The survey included demographic data, eating behaviours, psychological distress and both physical and psychosocial health-related quality of life.

**Results:**

The prevalence of possible ARFID was 1.98% (95% CI 1.63–2.41) and did not differ significantly across school years 7–12. The weight status of participants with possible ARFID did not differ significantly from those without possible ARFID. When measuring gender identity, the ratio of males to females with possible ARFID was 1:1.7. This was statistically significant, however, the effect size was very small. Psychological distress and HRQoL did not differ significantly between the possible ARFID and non-ARFID group.

**Conclusions:**

The prevalence of possible ARFID was found to be similar to that of anorexia nervosa and binge eating disorder in the general adolescent population. Adolescents who identify as girls rather than boys may be more likely to develop ARFID, replication with new samples is required to confirm these findings. The impact of ARFID on HRQoL may be minimal in adolescence and become more significant in adulthood, further research using longitudinal design, healthy control groups and/or diagnostic interviews is required.

## Abstract (plain language summary)

Little is known about how common and what the associated harms are for Avoidant/Restrictive Food Intake Disorder (ARFID) in community adolescent populations. In adult community populations, ARFID has been associated with impaired mental health and quality-of-life. This study aimed to investigate how common possible ARFID is and associated health-related quality of life and psychological distress in a sample of adolescents from the general population in New South Wales, Australia. From a sample of 5072 adolescents aged between 11 and 19 years of age we found 1.98% of adolescents had possible ARFID. The weight status of participants with possible ARFID did not differ from those without possible ARFID. When measuring gender identity, the ratio of males to females with possible ARFID was 1 to1.7. Psychological distress and quality of life did not differ significantly between the possible ARFID and non-ARFID group. From study results we could determine possible ARFID was as likely as anorexia nervosa and binge eating disorders in the general adolescent population. Adolescents who identify as girls rather than boys may be more likely to develop possible ARFID. The impact of ARFID on quality of life may be minimal in adolescence and become more significant in adulthood. Further research is needed to investigate this possibility.

## Background

Avoidant/restrictive food intake disorder (ARFID) was introduced in the Diagnostic and Statistical Manual for Mental Disorders, Fifth Edition (DSM-5) as the persistent failure to meet dietary needs for reasons other than fear of gaining weight, body image concerns, medical or cultural reasons [1]. Three primary reasons for restrictive eating behaviour in ARFID have been identified as [1] a lack of interest in food [2]; avoidance of sensory characteristics of food and [3] concern about aversive consequences of eating, such as choking [1]. To be diagnosed with ARFID, the restrictive eating behaviours must lead to either significant malnutrition, weight loss, compromised growth, dependence on supplements or enteral feeding, or impaired psychosocial functioning [1–3].

Neurobiological models of ARFID state that underlying the three primary profiles of ARFID are abnormalities in appetite regulation, sensory perception, and hyperactivation of the fear system [4]. This neurobiological abnormality and associated hyperactivation of the fear system may also contribute to comorbid mental health impairments in people with ARFID, including psychiatric comorbidity with anxiety disorders [4]. Unhelpful avoidance of feared stimuli may maintain food-related anxiety and lead to the development of chronic restrictive eating behaviours as seen in ARFID. Hence, ARFID represents a heterogenous group of individuals who vary significantly in the aetiology and maintenance of their restrictive eating behaviours, which appear to be the result of biological and environmental factors [5].

In clinical populations, ARFID has been associated with impaired psychosocial functioning [6, 7] and a wide array of physical health impairments, such as abdominal pain [3], electrolyte abnormalities [8], vitamin and mineral deficiencies [9] and amenorrhea in older girls [10]. Compared to individuals with anorexia nervosa, those with ARFID may experience chronic rather than acute weight loss [11], with earlier onset of disordered eating behaviour [12] and longer length of illness [13, 14], although it is also noted that individuals with ARFID who have an aversive consequences presentation can present with acute weight loss [15]. Aside from the anxiety disorders, ARFID also co-occurs commonly with neurodevelopmental conditions such as attention-deficit-hyperactivity-disorder and autism [16–21].

In non-clinical populations, ARFID has been associated with impaired mental health related quality-of-life in adults [21, 22] and impaired social functioning in children [23]. Despite growing evidence of the physical and psychosocial impairment associated with ARFID, the prevalence of ARFID remains unclear with wide-ranging estimates. In clinical samples, ARFID prevalence estimates have been reported between 1.5 and 64%—these samples have been recruited from a diverse range of settings including eating disorder clinics, paediatric hospitals, medical outpatient programs, and gastroenterology clinics. In community-based studies, reported ARFID prevalence estimates have also ranged considerably, from 0.3 to 15.5% [21, 24–28]. A consequence of the lack of clarity around the prevalence and impact of ARFID has been that ARFID is often excluded from public healthcare policies for eating disorder treatment. For instance, individuals with ARFID in Australia are not eligible to access a government supported Medicare benefit scheme specific for the treatment of other eating disorders, and which heavily subsidises appointments with dietitians, psychologists, and paediatricians [29]. Until further research can clarify the prevalence of ARFID in the general population and the impairment it causes, healthcare services and government bodies may continue to underestimate the need to support its treatment and prevention. While previous research on the prevalence of ARFID in the general population has examined samples of younger children [24–26, 28] and adults [21, 22, 27], to date there has been no research on the prevalence of ARFID among adolescents. This is a particularly important risk group to examine given that it is represents the peak period of risk for the development of other eating disorders [23] On the other hand, evidence suggests that unlike eating disorders, ARFID has an onset that typically occurs during childhood [12]. Thus it will be important to contrast the prevalence of ARFID and its prevalence distribution across early to late adolescence to the prevalence of other eating disorders during adolescence [30].

The present study aims were to investigate the prevalence and distribution of ARFID across weight, age and gender in a general adolescent population in Australia. The study also examined whether individuals with ARFID experience greater quality-of-life impairment and psychological distress compared to those without ARFID. In line with previous research, it was expected that individuals with ARFID would be more likely to present with an underweight BMI compared to individuals not meeting criteria for ARFID [11]. However, we did not expect ARFID to be associated with adolescent age, given reports of the onset of ARFID occurring primarily during childhood [12] and enduring for a longer course than other eating disorders [13, 14] This is expected to differ from the prevalence distribution pattern of other eating disorders during adolescence where a peak is typically observed in mid-late adolescence [30]. Gender was also not expected to emerge as a significant correlate of prevalence, given previous research in community populations has reported largely equal distributions of ARFID across male and female groups [25–28]. Based on previous findings summarized above [22], ARFID was hypothesized to be associated with lower psychosocial quality-of-life but similar physical health related quality-of-life. Further, since ARFID is highly comorbid with anxiety and neurodevelopmental conditions [16–21], it was hypothesised that individuals with ARFID would score higher on measures of psychological distress, compared to individuals not meeting criteria for ARFID.

## Method

The EveryBODY survey reported in this paper is part of a longitudinal investigation of eating disorders and body image concerns among Australian adolescents. The present study uses data from the first wave of the EveryBODY survey which involved a representative sample of students from 13 schools in the Newcastle and Sydney regions of Australia who completed the survey online in 2017.

### Participants and sampling

The current sample included 5072 adolescents aged between 11 and 19 years of age. Initially, 50 schools in the Newcastle and the Greater Hunter region of New South Wales were invited to participate. While eighteen schools initially agreed to participate, six schools withdrew prior to data collection. Schools that did not participate in the study outlined various reasons such as conflicting commitments, participation in other research projects, and lack of time or staff to facilitate the project. To increase ethnic diversity of the sample, seven schools from Sydney were invited to participate, and one agreed. A total of 13 schools (9 government-funded, 4 independent/private) participated in the current study.

The survey included questions on participant demographic characteristics, eating behaviours, weight and shape concerns, general mental health, and quality of life. In this first wave of the EveryBODY study 5191 students completed the survey. However, 119 students’ responses were excluded from the sample, for the following reasons: completing less than 10% of the survey (n = 39), for providing inappropriate/non-serious responses (n = 79), and for withdrawing consent (n = 1). For this specific study, a further *n* = 176 participants were excluded due to not completing the questions required to determine a possible ARFID diagnosis. This left a total study sample of *N* = 4896 students aged between 11 and 19 years.

At the time of the survey, 41.9% (*n* = 2051) of participants were in years 7–8, 40% (*n* = 1956) in years 9–10 and 18.2% (*n* = 889) in years 11–12; 46.9% (n = 2297) indicated a male sex, and 53.1% (*n* = 2599) indicated a female sex; 46.1% (*n* = 2052) identified their gender as male, 53.1% (*n* = 2364) as female, and 0.8% as non-binary (*n* = 35). Australia was the most common country of birth (89.5%, *n* = 4383), with a minority born in Asia (5.5%, *n* = 271) Europe (2.1%, *n* = 103), Oceania/Pacific Islands (1.2%, *n* = 60), Africa (0.9%, *n* = 43), North America (0.6%, *n* = 27) and South America (0.1%, *n* = 4). The overall socio-economic status (SES) of each school was estimated using the Index of Community Socio-Educational Advantage (ICSEA, standardized M = 1000, SD = 100), which is based upon geographic location, proportion of indigenous enrolments, and parental occupational and education. According to the ICSEA scores of schools in this sample, the average SES was similar to Australian schools in general, however with overall less variability.

### Ethics statement

Consent for participation in the survey was three-pronged. This involved the school’s consent as detailed above, the parents’ consent, and the student’s assent. Parents were given letters distributed by the school up to 4 weeks in advance about the upcoming EveryBODY survey and were invited to seek more information or opt their child out of participating. Parents’ consent was assumed unless they actively opted their child out of the study. On the day of testing, students provided assent before completing the survey online under the supervision of their teacher. After completion of the survey, students were given a handout outlining eating disorder specific and general mental health referral resources and support lines. Students also had the option of putting their name into a draw to win one of 10 $100 vouchers. Ethics approval was received from the University ethics committee, the Catholic Education Office, and the New South Wales Department of Education.

### Measures

#### Demographic characteristics

Participants self-reported their age in years and months, their biological sex (male or female), and their gender identity (male, female or other). One school did not allow presentation of a gender response option other than male or female. Participants also reported their subjective height and weight measurements which was used to calculate body mass index (BMI; weight (kg)/height (m) in accordance with the CDC guidelines (Centers for Disease Control and Prevention, 2017). BMI was then converted to BMI percentile, adjusting for sex and age. BMI percentile was classified according to the CDC classification scheme: above the 95th as obese, 85th to 95th percentile as overweight, 5th to 85th percentile as healthy weight, and under 5th percentile as underweight [31]. Self-reported height and weight measurements in adolescents are strongly correlated to their true anthropometric measurements [32].

#### ARFID diagnostic group

Responses to questions in the survey were used to determine a possible ARFID diagnosis based on the DSM-5 criteria for ARFID. All participants were asked "*are you currently avoiding or restricting eating any foods to the degree that you have lost a lot of weight or become lacking in nutrition (e.g., have low iron) or had problems with family or friends?*". Response options included: “*yes, because I dislike some foods, have a fear of swallowing or another reason*” (DSM-5 Criterion A), “*yes, for cultural reasons (e.g., Lent, Ramadan)*” (DSM-5 Criterion B), “*yes, for medical reasons (e.g., food allergy)*” (DSM-5 Criterion D), “*yes, I am dieting to prevent weight gain*” (DSM-5 Criterion C), and “*no, I am not avoiding or restricting eating any foods to that extent*”. If participants selected “*yes, because I dislike some foods, have a fear of swallowing or another reason*”, they were then asked to outline their reasons for avoiding or restricting food in an open-ended format. The open-ended responses were screened independently by two members of the research team (LVB and DM) to assess whether they met criteria for the reasons for food restriction in ARFID or not. Where there was disagreement in rating responses, a consensus was reached via discussion with a third researcher (CF).

A “possible ARFID” diagnosis was assigned to a participant if they met the following research criteria:

- Responded “*yes, because I dislike some foods, have a fear of swallowing* *or* *another* *reason*” to the question about food restriction above and not to one of the other medical/cultural/body image options which represent exclusion criteria for ARFID (DSM-5 Criterion A, B, D),
- Did not at the same time meet research criteria for anorexia nervosa or bulimia nervosa (diagnosis determined as published in a previous study on eating disorder prevalence using this sample [30]
- Scored below the established clinical cut-off (< 4) on the combined Weight and Shape Concern subscales of the Eating Disorder Examination [1, 30].
- Their stated reasons for restricting food intake aligned with ARFID criteria.

#### General psychological distress

The Kessler Psychological Distress Scale (K-10) was used as a measure of psychological distress experienced in the past 4 weeks. Participants answered 10 questions about the frequency and presence of symptoms characteristic of anxiety and/or depression on a five-point Likert-type scale, from “none of the time” [1] to “all of the time” [5]. Scores range from 10 to 50, with scores of 30 or higher indicative of severe distress. The K-10 has demonstrated high validity and internal consistency in general population samples, including with adolescents [33, 34]. In the present study, the K10 demonstrated excellent internal consistency with a Cronbach’s alpha of 0.94.

#### Health-related quality of life

Health-related quality of life (HRQOL) was measured using the 12 items from the physical functioning, emotional functioning, and social functioning subscales of the Pediatric Quality of Life Inventory (PedQL) [35]. The emotional and social functioning scales were combined to create a psychosocial subscale. The items asked participants to rate how true a series of statements are of them in the past 4 weeks, on a Likert type scale. Scores are reversed and transformed on a 0–100 scale, where higher scores indicate better HRQOL. In the present study, the physical functioning subscale and psychosocial subscale of the PedQL demonstrated good internal reliability with Cronbach’s α of 0.85 and 0.90, respectively.

#### Weight and shape concerns

Body weight and shape concerns were assessed using the combined weight and shape subscales of the Eating Disorder Examination Questionnaire (EDE-Q); [36]. The EDE-Q measures eating disorder pathology, and the frequency and severity of weight/shape concerns in the past 28 days on a seven-point Likert scale, from “no days/not at all” to “everyday/markedly” [36]. The EDE-Q weight and shape concerns subscale has demonstrated good reliability in adolescent samples from the general population [37]. In the present study, the EDE-Q subscale demonstrated excellent internal reliability with Cronbach’s α of 0.96.

### Data analysis

Data were explored using analysis from the statistical software package SPSS (version 22, 2014; SPSS Inc, Chicago, IL, USA). To determine outcomes for the first aim, we calculated the point prevalence of possible ARFID in the overall sample with 95% confidence interval. Next, to understand whether the distribution of possible ARFID varied across demographic characteristics, chi-square analyses were conducted to compare prevalence of ARFID across weight status categories (underweight, healthy weight, overweight and obese), year groups (year 7–8, 9–10, and 11–12) and gender (male, female and other). To assess relative contributions of these demographic variables to possible ARFID prevalence, weight status, gender, and year group were entered as predictor variables into a multivariate binomial logistic regression with possible ARFID as the outcome variable. No issues with multicollinearity were detected. An inspection of z-scores on a boxplot indicated that no outliers were present for gender and year group, but several outliers were detected for weight status. These outliers were participants who had been categorised as CDC underweight, overweight, and obese (vs “healthy weight”). As a lower prevalence of underweight, overweight, and obese is typical of a community adolescent population [38], these outliers were retained for analyses.

For the second aim, to determine the association between possible ARFID and indicators of impairment, three univariate ANOVAs were conducted with ARFID as the predictor variable and psychological distress, physical HRQoL and psychosocial HRQoL as outcome variables. Univariate ANCOVAs were then conducted while adjusting for BMI percentile, gender, and age, due to their independent impact on psychological functioning and QoL in adolescence (Bisegger et al., 2005; Fallon et al., 2005). The assumptions for univariate ANCOVA’s were met for all three outcome variables (psychological distress, physical HRQoL and psychosocial HRQoL), except for the standardized residuals which were not normally distributed according to Shapiro–Wilk’s test (*p* < 0.05). However, due to the large sample size, the univariate ANCOVA can be considered robust, and non-normality does not affect Type 1 error rate substantially [39]. While two-way ANCOVA’s would have allowed for analysis of the interactions between possible ARFID and demographic characteristics (weight status, gender, and year group), heterogeneity of variance meant that statistical assumptions were violated, and were deemed inappropriate to use in this case.

## Results

### Prevalence

When applying research criteria a, b and c above for possible ARFID, *n* = 144 participants were identified. However, after applying criterion d, 47 of these participants were excluded. Reasons for exclusion included that participant avoided or restricted food intake for non-ARFID related reasons such as for cultural reasons (*n* = 1), weight and shape concerns (*n* = 13), other dietary reasons such as to be healthy or concern for animal cruelty (*n* = 20), or because participants provided nonsensical responses (*n* = 2) or no reason at all for their food restriction/avoidance (*n* = 11). This left a final sample of *n* = 97 participants who were operationally categorised as meeting criteria for possible ARFID and a point prevalence of 1.98% (95% CI 1.63 to 2.41%). Table 1 further breaks down the prevalence within demographic groups. Coding of the open-ended responses for the reasons participants’ provided for their food restriction/avoidance found that the vast majority reported their reason was a dislike of the taste or texture of certain foods (*n* = 75; 77%). Other reasons included fear of aversive consequences such as vomiting or becoming unwell (*n* = 9; 9%), and lack of appetite or interest in eating (*n* = 7; 7%). A further 4 participants reported that they did not know why they restricted or avoided foods, and two responses were vague (“because I can”, “sometimes”).

**Table 1:** Prevalence of ARFID within gender, age, and weight status group

	Possible ARFID % (*n*)	No ARFID % (*n*)	95% CI	χ^2^ (*df*)	Cramers V
*Gender*					
Boys	1.4 (29)	98.6 (2023)	0.98–2.02	6.27 (2)	0.038
Girls	2.5 (58)	97.5 (2306)	1.90–3.16		
Other	2.9 (1)	97.1 (34)	0.51–14.53		
*Age*					
Year 7–8	2.3 (48)	97.7 (2003)	1.76–3.09	2.58 (2)	0.023
Year 9–10	1.6 (32)	98.4 (1924)	1.16–2.30		
Year 11–12	1.9 (17)	98.1 (872)	1.20–3.04		
*BMI*					
< 5th percentile	3 (11)	97 (358)	1.67–5.25	2.95 (3)	0.026
5–85th percentile	2.1 (68)	97.9 (3104)	1.69–2.70		
85–95th percentile	1.4 (9)	98.6 (613)	0.76–2.72		
> 95th percentile	2.6 (9)	97.4 (343)	1.35–4.78		

Within the group with possible ARFID (and with rounding), 67% identified their gender as female, 33% as male, and 2.9% as non-binary/non-conforming “other” gender (vs. 53%, 46%, and 1% in the non-ARFID group, respectively). In terms of school grade, 50% were currently in years 7 or 8 (early-adolescence), 33% were in year 9 or 10 (mid-adolescence), and 18% were in years 11 or 12 (late-adolescence; vs. 42%, 40%, and 18% in the non-ARFID group, respectively). In regards to CDC weight categories, 11% were classified as “underweight”, 70% as “healthy weight”, 9% as “overweight”, and 9% as “obese” (vs. 8%, 70%, 14%, and 8% in the non-ARFID group, respectively).

#### Univariate effects of gender, age and weight on prevalence

Table 1 displays the prevalence of ARFID across gender, weight status and year groups.

#### Gender

The overall effect of gender was significant. Girls were statistically more likely than boys to meet criteria for possible ARFID (*p* = 0.016, Cramer’s *V* = 0.037). Although there was a trend for ARFID to be highest among the non-binary gender group, the rate compared to boys (*p* = 0.400) or girls (*p* = 0.584) was not statistically significantly different.

#### Age

The prevalence of possible ARFID did not vary significantly across age groups (*p* = 0.275, Cramer’s *V* = 0.023), although there was a trend for possible ARFID to be more common among early adolescent participants in years 7–8 (2.3%), compared to older adolescents in years 9–10 (“middle adolescence”; 1.6%) and 11–12 (“late adolescence”; 1.9%).

#### Weight status

The prevalence of possible ARFID was not statistically significantly associated with weight status categories (*p* = 0.399, Cramer’s V = 0.026). However, a trend emerged whereby the highest prevalence of possible ARFID was found in participants classified as "underweight” (3.0%) or “obese” (2.6%) compared to the middle categories of “healthy” or “overweight” (2.1% and 1.4%, respectively).

#### Multivariate analysis of demographic correlates of ARFID prevalence

A binomial logistic regression was performed to ascertain the independent effects of age, weight status and gender on the likelihood that participants met criteria for possible ARFID. The model was statistically significant, χ^2^(7) = 14.251, *p* = 0.047. Of the three predictor variables, only gender was significant (*p* = 0.018), where girls had 1.72 (95% CI 1.10–2.17) higher odds of meeting criteria for possible ARFID compared to boys.

### Impairment and distress associated with ARFID

Table 2 displays a summary of quality of life and psychological distress scores among participants with vs without possible ARFID.. The unadjusted ANOVAs examining the association of possible ARFID with physical HRQoL scores (*F*(1, 4340) = 0.08, *p* = 0.778, partial η^2^ = 0.000), psychosocial HRQoL scores (*F*(1, 4340) = 2.236, *p* = 0.135, partial η^2^ = 0.000), and psychological distress scores (*F*(1, 4701) = 2.272, *p* = 0.132, partial η^2^ = 0.000) however found no effects of possible ARFID on scores. This remained the case after adjustment of the ANCOVA models for age, sex and weight status [(*F*(1, 3620) = 0.109, *p* = 0.741, partial η^2^ = 0.000); (*F*(1, 3620) = 1.723, *p* = 0.189, partial η^2^ = 0.000); (*F*(1, 3916) = 1.752, *p* = 0.186, partial η^2^ = 0.000), respectively]. Overall, these results suggest that possible ARFID in this sample was not significantly associated with elevated psychological distress or impairment in physical or psychosocial HRQoL.

**Table 2:** Means and standard deviations for health-related quality of life and psychological distress scores for participants identified with versus without possible ARFID

	Physical HRQoL M (*SD*)	Psychosocial HRQoL M (*SD*)	Psychological distress M (*SD*)
*Unadjusted*			
ARFID	85.33 (17.20)	73.03 (21.71)	22.47 (8.90)
Non-ARFID	85.90 (18.89)	76.73 (23.15)	20.89 (10.14)
*Adjusted* *for* *gender,* *weight,* *age*			
ARFID	85.00 (17.82)	72.68 (22.3)	22.55 (9.00)
Non-ARFID	85.63 (19.13)	76.43 (23.30)	21.04 (10.20)

ARFID, avoidant/restrictive food intake disorder; HRQoL, health-related quality of life; Physical and Psychosocial HRQoL measured using the Pediatric Quality of Life Scale; Psychological distress measured using the Kessler Psychological Distress Scale

As a post-hoc analysis, to further explore impairment and distress associated with possible ARFID, the prevalence of possible ARFID was recalculated when an additional “clinical significance” criterion was added. This criterion does not appear in the formal DSM-5 diagnosis of ARFID, although it is a common criterion in most other DSM-5 mental disorders. The operationalization was borrowed from the study of eating disorder prevalence using the same sample [30]: a score of 30 or higher on the K-10 and/or scoring at least one SD below the sample mean on the physical or psychosocial HRQoL score. When this clinical significance criterion was applied, the prevalence of possible ARFID dropped by a little over two thirds from 1.98% (*n* = 97) to 0.76% (*n* = 37). This new group of 37 participants with possible ARFID associated with distress and/or impairment were on average 14.8 years (SD = 1.7); 25 were girls, 11 were boys, and 1 was agender; and 14% had a BMI percentile classified as underweight, 73% as healthy, 3% as overweight and 11% as obese.

## Discussion

The present study aimed to investigate the prevalence and impairment associated with possible ARFID in a community adolescent population. The prevalence rate of possible ARFID in this population was 1.98%, which is within the range reported by previous studies that applied similar diagnostic criteria to assess the prevalence of ARFID in general child populations (0.35–3.2%; [24, 28] and adult populations globally (0.3–3.1%; [21, 22, 27]. The prevalence of ARFID appears to be similar to other eating disorders in the same sample populations, such as anorexia nervosa (0.5%), bulimia nervosa (3.3%) and binge eating disorder (0.8%) [30].

In contrast to our hypothesis, the weight status of participants with ARFID did not differ significantly to those without possible ARFID. Although, the highest rates of possible ARFID were observed in both the “underweight” and “obese” weight groups. This finding is consistent with the DSM-5 diagnostic conceptualisation of ARFID, which does not necessitate significant weight loss and emphasises the varied nutritional and functional impact of ARFID on the individual [1]. It is noted that adolescents in the community with higher weights are known to be more at risk for almost all eating disorders, including restrictive phenotypes (with the exception of anorexia nervosa) [30]. These findings indicate that, as with other eating disorders [40], weight should not be relied upon when screening for possible ARFID adolescent populations. On the other hand, as with other eating disorders, people with ARFID in treatment settings are more likely to be underweight, and this may be due to significant weight loss being a powerful prompter for referral and treatment-seeking [41].

Consistent with our hypotheses was the finding that the prevalence of ARFID did not differ across age groups. This supports previous findings that the onset of ARFID is predominantly in childhood [12]; and that ARFID appears to have a more stable chronic and long-term trajectory compared to other eating disorders, which appear to peak in mid-to-late adolescence and be associated with a more variable course [13, 14].

Contrary to our hypothesis, the present study found that girls were 1.7 times more likely to be identified by our study criteria for ARFID compared to boys. However, the present study explored the prevalence of ARFID across groups based on gender rather than sex assigned at birth. This is in contrast with previous research in community samples that measured sex assigned at birth and found equal distribution of ARFID diagnoses between males and females [25–28]. However, the effect size of this finding was very small, and more research is required to understand the relationship between gender, assigned sex, and ARFID. Moreover, girls may be more likely to disclose mental health problems on self-report measures than boys, including in responses to an online survey such as implemented in this study [42].

The present study found no significant difference between individuals with and without possible ARFID on physical health related quality of life, which is consistent with previous research in community populations [22]. Contrary to our hypothesis, however, is the finding that individuals identified with possible ARFID also scored similarly to those without possible ARFID on measures of psychosocial quality of life and psychological distress. This contrasts with findings that ARFID is highly comorbid with anxiety disorders [20, 21] and is associated with impaired social and emotional quality of life [21, 22]. This may suggest that quality of life impairment becomes more significant the longer someone is impacted by ARFID, as previous studies have shown psychosocial quality of life impairment in adults with ARFID, whereas the present study examined an adolescent population [21, 22]. In keeping with this hypothesis, another previous study conducted with a sample of children identified with ARFID found no impairment in psychological and physical well-being relative to a control group [23]. On the other hand, this study did find that children with ARFID had relative deficits in peer relations and autonomy from their parents—which may indicate that these are either risk factors or important early indicators of ARFID impairment in younger age groups. On the other hand, around one third of the participants identified in our study with possible ARFID did also meet a post-hoc applied “clinical significance” criterion based on their endorsement of either severe distress or impairment in physical or psychological HRQoL. The prevalence of this subgroup of possible ARFID with clinical impairment was 0.76% and may come closer to reflecting the nature and prevalence of adolescents with ARFID who present to clinical settings. Greater awareness at a community and clinical level may be needed to ensure that these children are identified and able to access early intervention to reduce the chance of prolonged adverse health outcomes.

## Strengths and limitations

A strength of the present study is the exclusion of participants from the possible ARFID group who had reported significant weight and shape related body image concerns, or reported medical, cultural, or other dietary reasons for their restrictive eating behaviour. Moreover, participants who reported restrictive eating behaviours were prompted to provide reasons for this behaviour in an open-ended response format that was then screened by researchers for allocation to the possible ARFID or non-ARFID group. However, a limitation is that the participants’ reported reasons for food restriction often did not provide enough information to make accurate diagnostic decisions. For example, participants appeared to sometimes misunderstand the question, did not provide an answer, or provided vague information that was left up to subjective interpretation. This may have led to the exclusion of genuine cases of possible ARFID, and the inclusion of non-ARFID cases in the diagnostic group. A clinical interview that allows for follow-up questions and clarification would have facilitated more accurate diagnostic decisions. Also related to ARFID operationalization is the complexity of the initial screening question (participants were asked to identify with both the restrictive/avoidant food intake behaviour as well as the negative consequences of this behaviour), which had the potential to be misunderstood.

A limitation that may have contributed to the unexpected findings regarding impairment is the presence of individuals with other eating disorder and mental health diagnoses in the non-ARFID group. A previous epidemiological study by Mitchison and colleagues (2019) found a point prevalence of 22.2% for any eating disorder (including 0.7% for anorexia nervosa, 4.6% for probable bulimia nervosa, and 1.0% for probable binge-eating disorder) in the present study’s sample of adolescents. These eating disorders were not screened out of the comparison (non-ARFID) group in the present study and given their known association with mental health impairment [22, 43–45], this may partly explain the lack of group differences we observed. It is also reasonable to assume that the non-ARFID group contained participants with other mental health disorders, as epidemiological research on a general sample of adolescents in Australia found that 14.2% had depression and 13.2% had anxiety [46]. On the other hand, comorbid mental health disorders were just as likely, if not more so, to be present in the possible ARFID group. Capturing and controlling for comorbid psychiatric illness will be helpful in future study designs to reduce type 2 error when examining impairment associated with ARFID.

Another limitation is that although we could describe participant’s self-reported reasons for their ARFID behaviour (i.e., due to taste and texture aversion; fear of aversive consequences etc.), we could not statistically examine the association between reasons for ARFID behaviour and outcomes of interest. Future population-based studies should prioritise recruitment of larger samples, to facilitate such research, especially as it is possible that the preponderance of self-reported sensory reasons for ARFID behaviour may in part explain the relationship observed with weight in this study, if for instance sensory avoidance did not lead to an overall caloric restriction for some individuals. Relatedly it would be important for future studies to examine other indicators of nutritional and medical impairment associated with reasons for ARFID behaviour.

## Clinical implications and future research directions

The prevalence of possible ARFID is similar to other eating disorders in the general adolescent population, however the impairment associated with ARFID appears to be milder in the community compared to clinical settings. The finding that quality of life and psychological distress did not differ significantly between adolescents with and without possible ARFID should be interpreted with caution due to the lack of a healthy control group. In conjunction with research that demonstrates psychosocial impairment in adults with ARFID, the present study’s findings may suggest that quality of life impairment emerges in adulthood, after the individual has experienced ARFID for a longer duration of their life. If this is the case, then public health care systems that allow for early detection and treatment at a community level or provide environments in which young people can seek and access help, will reduce the development of significant impairment in individuals with ARFID.

Due to the limitations of the present study, further research is required to understand the impairment associated with ARFID in community settings. A longitudinal study design would help to examine whether psychosocial quality of life impairment varies across the life span for individuals with ARFID. Moreover, future studies should capture and control for comorbid psychiatric illness in the control group to reduce the likelihood of overlooking significant impairment in the possible ARFID group. Future studies are also encouraged to utilise unstructured or semi-structured interviews with participants to ensure accurate diagnosis of ARFID in participants. Studies such as these would contribute to a greater understanding of the prevalence and impairment associated with ARFID, which is essential for the development of appropriate and accessible services for a global eating disorder.

## Conclusions

The present study is the first to explore the prevalence and burden associated with possible ARFID in a community adolescent population. The prevalence of possible ARFID is similar to other eating disorders in the general adolescent population, such as anorexia nervosa and binge eating disorder. Psychosocial quality of life appears to be intact in young people with possible ARFID, with impairments observed in studies with adults possibly emerging later in life. Health care systems that facilitate detection and treatment of ARFID in children and adolescents may help to reduce the development of significant psychosocial and quality of life impairment in adulthood. However, further research using healthy control groups, diagnostic interviews, and/or a longitudinal design, is needed to understand the impairment associated with ARFID across the lifespan.

## Data Availability

Data are available from the corresponding author for the purpose of secondary data analyses.

## Abbreviations

ARFID: Avoidant/restrictive food intake disorder
ANOVA: Analysis of variance
EDE-Q: Eating Disorder Examination Questionnaire
DSM5: Diagnostic and Statistical Manual for Mental Disorders, Fifth Edition
HRQoL: Health-related quality of life.
PedQL: Pediatric Quality of Life Inventory
