# Correction: The prevalence and burden of avoidant/restrictive food intake disorder (ARFID) in a general adolescent population

**DOI:** 10.1186/s40337-023-00873-1

**Published:** 2023-08-29

**Authors:** Lara Van Buuren, Catharine Anne Kerle Fleming, Phillipa Hay, Kay Bussey, Nora Trompeter, Alexandra Lonergan, Deborah Mitchison

**Affiliations:** 1 School of Psychology, Western Sydney University, Sydney, Australia; 2 School of Health Sciences, Western Sydney University, Sydney, Australia; 3 Translational Health Research Institute, School of Medicine, Western Sydney University, Sydney, Australia; 4 Centre for Emotional Health, School of Psychological Sciences, Macquarie University, Sydney, Australia

**Correction: J Eat Disord 11, 104 (2023)**. 10.1186/s40337-023-00831-x

Following publication of the original article [1], the authors reported that the review process was mistakenly included in the article.

The review process has been removed and the original article [1] has been updated.
